# Prevalence of *ABCA4* Deep-Intronic Variants and Related Phenotype in An Unsolved “One-Hit” Cohort with Stargardt Disease

**DOI:** 10.3390/ijms20205053

**Published:** 2019-10-11

**Authors:** Marco Nassisi, Saddek Mohand-Saïd, Camille Andrieu, Aline Antonio, Christel Condroyer, Cécile Méjécase, Juliette Varin, Juliette Wohlschlegel, Claire-Marie Dhaenens, José-Alain Sahel, Christina Zeitz, Isabelle Audo

**Affiliations:** 1 Sorbonne Université, INSERM, CNRS, Institut de la Vision, F-75012 Paris, France; m.nassisi@gmail.com (M.N.); saddekms@gmail.com (S.M.-S.); aline.antonio@inserm.fr (A.A.); christel.condroyer@inserm.fr (C.C.); cecile.mejecase@inserm.fr (C.M.); juliette.varin@inserm.fr (J.V.); juliette.wohlschlegel@inserm.fr (J.W.); j.sahel@gmail.com (J.-A.S.); 2 Centre Hospitalier National d’Ophtalmologie des Quinze-Vingts, INSERM-DHOS CIC 1423, F-75012 Paris, France; candrieu@15-20.fr; 3 Univ Lille, Inserm UMR-S 1172, CHU Lille, Biochemistry and Molecular Biology Department-UF Génopathies, F-59000 Lille, France; claire-marie.dhaenens@chru-lille.fr; 4 Fondation Ophtalmologique Adolphe de Rothschild, F-75019 Paris, France; 5 Académie des Sciences-Institut de France, F-75006 Paris, France; 6 Department of Ophthalmology, The University of Pittsburgh School of Medicine, Pittsburg, PA 15213, USA; 7 Institute of Ophthalmology, University College of London, London EC1V 9EL, UK

**Keywords:** *ABCA4*, Stargardt disease, deep-intronic variants, genotype-phenotype correlation

## Abstract

We investigated the prevalence of reported deep-intronic variants in a French cohort of 70 patients with Stargardt disease harboring a monoallelic pathogenic variant on the exonic regions of *ABCA4*. Direct Sanger sequencing of selected intronic regions of *ABCA4* was conducted. Complete phenotypic analysis and correlation with the genotype was performed in case a known intronic pathogenic variant was identified. All other variants found on the analyzed sequences were queried for minor allele frequency and possible pathogenicity by *in silico* predictions. The second mutated allele was found in 14 (20%) subjects. The three known deep-intronic variants found were c.5196+1137G>A in intron 36 (6 subjects), c.4539+2064C>T in intron 30 (4 subjects) and c.4253+43G>A in intron 28 (4 subjects). Even though the phenotype depends on the compound effect of the biallelic variants, a genotype-phenotype correlation suggests that the c.5196+1137G>A was mostly associated with a mild phenotype and the c.4539+2064C>T with a more severe one. A variable effect was instead associated with the variant c.4253+43G>A. In addition, two novel variants, c.768+508A>G and c.859-245_859-243delinsTGA never associated with Stargardt disease before, were identified and a possible splice defect was predicted *in silico*. Our study calls for a larger cohort analysis including targeted locus sequencing and 3D protein modeling to better understand phenotype-genotype correlations associated with deep-intronic changes and patients’ selection for clinical trials.

## 1. Introduction

Stargardt disease (STGD1; MIM #248200) is the most common juvenile macular dystrophy, with a prevalence of 1 over 8.000–10.000 [1]. It is an autosomal recessive disease, associated with mutations in *ABCA4* (MIM #601691) [2]. This disease affects only the retina and leads to a progressive loss of retinal structure and function. Although it affects only the retina, it is a clinically heterogeneous disease. Indeed, the phenotype could range from a severe involvement of the macula with diffuse atrophy, generalized retinal dysfunction, and visual impairment at early ages to a milder late onset disease (after the age of 50) with foveal sparing and relative preservation of the visual function. Symptoms at onset include progressive bilateral visual acuity reduction associated with dyschromatopsia (impairment of the color vision). Fundus examination shows progressive central atrophy with yellowish flecks at the level of the retinal pigment epithelium (RPE) (the single cell layer between the retina and the underlying choroid). The age of onset is usually during childhood, with a second peak in adulthood (around 20 years) and a less common late-onset STGD1 after 50. The latter usually has a better visual prognosis. Nowadays, there is mounting evidence that the heterogeneity of the phenotype is related to the severity of the genetic variants on *ABCA4*, with a loss of function leading to an earlier onset and a faster progression, while later onset with foveal sparing are usually associated with milder variants [3,4]. Since an important phenotypic variability between and within families has also been described, it was suggested that other factors, including genetic and environmental factors, can influence the phenotype [5].

*ABCA4* is located on chromosome 1p22.1 and comprises 50 exons spanning about 150 kb of genomic sequence. It encodes the transmembrane protein, retinal-specific ABCA4, a member of the superfamily ATP-binding cassette (ABC) transporter which is composed of 2273 amino acids. This protein is localized at the level of the outer segment (OS) of cones and rods, and to some extent in the retinal pigment epithelium (RPE) and is involved in the translocation of retinoids across OS disc membranes prior to their active transport from photoreceptors to the RPE [6]. 

*ABCA4* is a highly polymorphic gene with more than 1000 reported disease causing variants. Many of them are not only associated with STGD1, but also with different phenotypes such as cone or cone-rod dystrophies or retinitis pigmentosa [7,8]. This polymorphic nature makes it challenging to identify the pathogenic variants and distinguish them from benign variants; furthermore, the allelic heterogeneity complicates the genotype-phenotype correlation. In general, missense variants are associated with a milder phenotype compared to nonsense or frameshift variants. However, there are frequent exceptions like the complex alleles p.[(Leu541Pro;Ala1038Val)] or p.(Arg1640Trp) which are known to cause severe phenotype.

Recently, the understanding of disease-causing genetic variations in *ABCA4* has substantially improved as a result of two major advances. First, several noncoding disease-associated *ABCA4* alleles have been identified and proven to be pathogenic, mostly by affecting splicing [9,10,11,12,13,14,15,16,17,18]. Second, it has been determined that some *ABCA4* variants, which were initially considered as benign because of their high frequency in the general population, are in fact very mild conditional alleles: only when these variants are *in trans* with a deleterious mutation, it results in disease expression. These variants are thereby called “extreme hypomorphs” [19]. Nevertheless, around 15% of STGD1 cases remain genetically «unsolved», with only one mutation detected despite precise genetic analysis. If we consider that large copy-number variants (CNVs), often overlooked by conventional Sanger sequencing, are exceptional in the *ABCA4* locus [20], the remaining “missing” alleles in monoallelic cases likely reside in the noncoding sequences of *ABCA4*. 

Few recent reports studied the prevalence of deep-intronic variants in large cohorts, but limited data were provided for genotype-phenotype correlation. Having a comprehensive genotype-phenotype correlation is however important, as it helps clinicians in predicting patients’ prognosis, in finding morphological or functional characteristics that may help following the evolution of the disease and eventually the response to a future treatment and finally in selecting the best subjects for ongoing and future therapeutic trials.

The aim of this study was to determine the prevalence of reported deep-intronic variants in a French cohort of 70 patients with a clinical diagnosis of STGD1, carrying only one pathogenic allele as determined previously by direct sequencing of the exonic and flanking parts of *ABCA4*. 

## 2. Results

### 2.1. Genotypic Analysis

This is a retrospective study based on the preliminary results of the genetic screening of an initial cohort of 528 subjects with a clinical diagnosis of STGD1, seen at the National Reference Center for Rare Retinal Diseases of the Quinze-Vingts Hospital, Referet, Paris, France. The initial screening on the exonic parts of *ABCA4* started in 2007 and was performed with methods available at our institution at that time, including microarray analysis and Sanger sequencing. This led to the identification of seventy index patients carrying only one pathogenic variant. These subjects and their siblings (when available) were screened for the presence of the 24 known *ABCA4* deep-intronic variants included from the review of the literature (Table S1). The second mutated allele was found in fourteen subjects (20%). The complete genotypic information and cosegregation analysis for these patients are reported in Table 1. Among the variants investigated, the most frequent was c.5196+1137G>A in intron 36 (6 subjects, all Europeans), followed by c.4539+2064C>T in intron 30 (4 subjects, 3 Europeans and 1 African) and c.4253+43G>A in intron 28 (4 subjects, all Europeans). Variant c.4253+43G>A has a minor allele frequency (MAF) of 0.00047, with the highest frequency among the Ashkenazi Jewish (0.0124), followed by Finnish Europeans (0.0096) and non-Finnish Europeans (0.006). Variant c.4539+2064C>T is absent in gnomAD while variant c.5196+1137G>A has a MAF of 0.00009 with the highest frequency among the non-Finnish European population of 0.0001. Twelve deep-intronic variants, which were never reported as associated with retinal diseases and with a MAF ≤ 0.01, were identified during the screening and further analyzed for conservation and *in silico* predictions (Table 2). Among those, 2 variants showed possible changes on splicing on the *in silico* predictions: c.768+508A>G and c.859-245_859-243delinsTGA (Figures S1 and S2). Variant c.768+508A>G (harbored by CIC03956) has a MAF of 0.0076 and is in a conserved *ABCA4* region for mammalians (except cow and tree shrew, Table S2). Two predictive algorithms reveal that it could cause an inactivation of a donor site and an activation of an acceptor site (Figure S1). The presence of a close strong donor site at c.768+804 (as predicted by all algorithms) might favor the insertion of a pseudoexon of 295bp between exons 6 and 7, causing a translation frameshift p.(Leu257Trpfs*24). Variant c.859-245_859-243delinsTGA (harbored by CIC01916) is not present in gnomAD and is located in a conserved region of the gene among primates (Table S2). This variant is predicted to create a strong donor site by all the algorithms used in the study (Figure S2). The presence of a close strong acceptor site at c.859-337 (as predicted by all algorithms) might favor the insertion of a pseudoexon of 91bp between exons 7 and 8, causing a translation frameshift p.(Phe287Argfs*14). As the co-segregation analysis was not possible and no functional tests were performed, these variants remain of unknown significance, in accordance with the American College of Medical Genetics recommendations [21]. Among the other detected novel variants, three of them (c.859-241A>C, c.1938-703C>T and c.4539+1168C>G) showed a moderate/high conservation among mammalians, together with a very low frequency (or even absent) in the general population. Even though no effects were predicted on splicing by the *in silico* analysis, we cannot completely exclude that they could indeed affect it in vivo, causing the disease. 

### 2.2. Phenotypic Analysis and Genotype-Phenotype Correlation

Charts from the 14 subjects harboring a deep-intronic variant were reviewed and all clinical data available collected. These data are presented in Table 3.

#### 2.2.1. c.4253+43G>A

**Figure 1:**
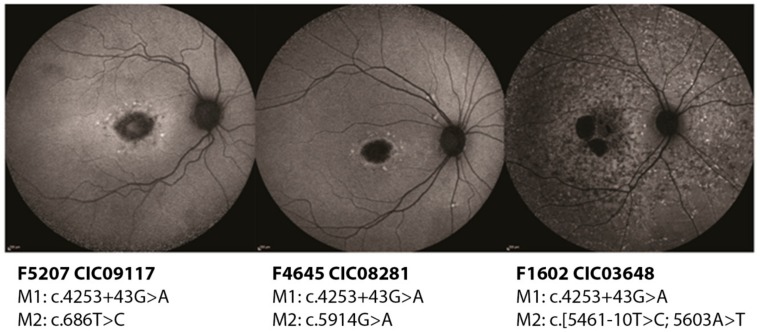
Short wavelength autofluorescence images of the right eyes of three patients carrying the deep-intronic variant c.4253+43G>A. While CIC09117 (47 years old) and CIC08281 (45 years old) show a milder phenotype with localized lesions, CIC03648 (26 years old) has clearly a more extensive disease.

Subjects CIC03648, CIC08281, CIC09117, and CIC09817 harbor the variant c.4253+43G>A on intron 28 (pedigrees shown in Figure S3). CIC09117, CIC08281 and CIC09817 show a milder phenotype with a later age of onset, peripapillary sparing, and intact photoreceptor function as assessed by the full-field electroretinogram (ff-ERG). Instead, CIC03648 shows a more severe phenotype with an earlier age of onset, presence of diffused flecks, and impairment of the cone responses on the ff-ERG. Based on these data, overall, a mild or hypomorphic effect of c.4253+43G>A could be hypothesized. This could be confirmed in family F5207 where CIC09119, unaffected father of the index patient CIC09117, is homozygous for the intronic variant, but shows no phenotype. CIC08281 harbors c.4253+43G>A with c.5914G>A showing a mild phenotype. Retaining the hypothesis of a hypomorphic effect of the intronic change, c.5914G>A should be considered severe. Indeed, c.5914G>A affects a highly conserved region of the second nucleotide binding domain (NBD) in ABCA4 and it has been demonstrated that missense variants involving one of the two NBDs tend to produce a more severe phenotype [4]. On the other hand, CIC03648 carries c.4253+43G>A together with the complex allele c.[5461-10T>C;5603A>T] [13]. This complex allele is known to be a severe allele with a loss of function of the protein. In this case, the presence of c.4253+43G>A should alleviate the expressed phenotype, which, instead, looks particularly severe, with an early age of onset and the presence of an extensive retinal disease (Figure 1). 

CIC09817 harbors c.4253+43G>A together with c.5603A>T, which is a well-known hypomorphic variant. As two hypomorphic variants should not be able to produce phenotype, it is possible that: (1) the two variants are in *cis* and a third severe variant on an unscreened region of *ABCA4* could be present (unfortunately no DNA from additional family members was available for this patient to verify the allele phase); (2) one of these two variants is in *cis* with a severe variant; (3) the effect of c.4253+43G>A might not be always mild or hypomorphic depending on the presence of another severe variant in *cis*.

#### 2.2.2. c.4539+2064C>T

**Figure 2:**
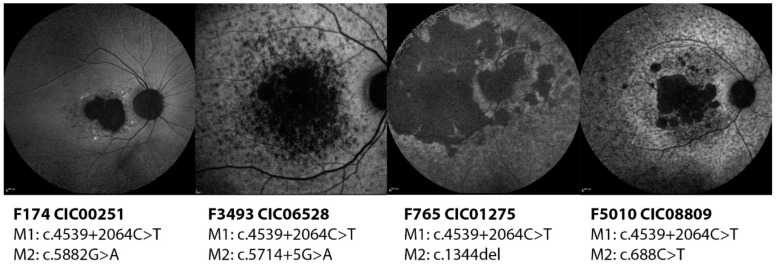
Short wavelength autofluorescence images of the right eyes of four patients carrying the deep-intronic variant c.4539+2064C>T. All phenotypes look quite advanced with foveal involvement and diffuse flecks and atrophy at the posterior pole except CIC00251, who carries the mild variant c.5882G>A.

Subjects CIC00251, CIC01275, CIC06528 and CIC08809 harbor the variant c.4539+2064C>T on intron 30 (pedigrees shown in Figure S4). All four show a severe phenotype with early onset disease, diffused flecks and photoreceptor impairment, in particular in subjects CIC01275 and CIC06528 who harbor a second severe variant (c.1344del and c.5714+5G>A respectively). CIC00251 carries the mild variant c.5882G>A which seems to mitigate the phenotype as this patient preserves a good color vision and a normal ff-ERG. Between these two “extremities”, CIC08809 has a relatively “intermediate” phenotype with a good residual function of the rods; this patient carries a missense variant c.688T>G which is located on the first extracellular domain (ECD) and might result in some residual activity of the protein, hence the phenotype (Figure 2).

#### 2.2.3. c.5196+1137G>A

**Figure 3:**
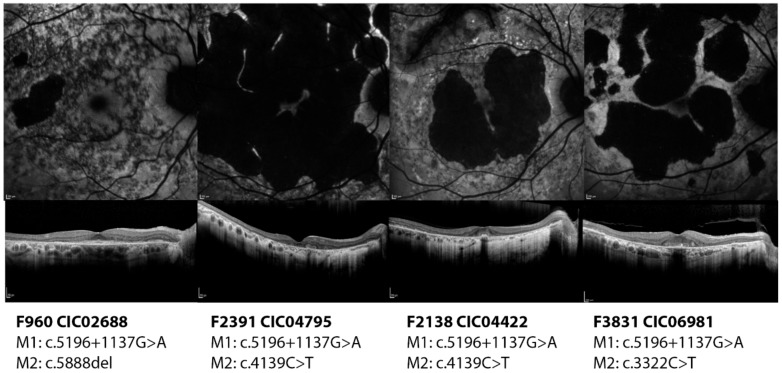
Short wavelength autofluorescence images and optical coherence tomography foveal scan of the right eyes of four patients carrying the deep-intronic variant c.5196+1137G>A. All four patients show a diffuse disease with macular atrophy. However, they all show foveal preservation which ensure them a relatively good residual visual acuity.

Subjects CIC02688, CIC10544, CIC04795, CIC06981, CIC07459, and CIC04422 harbor the variant c.5196+1137G>A on intron 36 (pedigrees shown in Figure S5). With the exception of CIC07459, the other subjects show a later disease onset with foveal sparing, which allows them to preserve a central area of vision with a relatively good visual acuity (Figure 3). 

It is reasonable to suppose that the effect of this variant could be mild; however, with the exception of CIC02688 who carries a severe variant, all other patients carry different missense variants, whose effect might be variably correlated to the phenotype. Indeed, CIC06981, CIC04422 and CIC04795 carry variants that are localized on an highly conserved region of the first NBD whose conformation is crucial for the correct function of the protein and whose alterations are associated with a more severe phenotype [4]. Even though they share the same age (72 years old), compared to CIC06981, the phenotype looks worse for CIC04422 who has a more extensive atrophy and impaired function of the photoreceptors. CIC07459 (carrying also the variant c.1804C>T) is the most severe among the subgroup, given that she already has central atrophy and generalized cone impairment at such a young age (22 years old). 

#### 2.2.4. Novel Variants

Variant c.768+508A>G was harbored by CIC03956 in *trans* with the missense variant c.1927G>A, p.(Val643Met). Unfortunately, as this patient was sampled many years ago and she did not undergo any ophthalmic examination in our center during the last 15 years, precise phenotypic data were not available. Variant c.859-245_859-243delinsTGA was harbored by CIC01916 in *trans* with the complex allele c.[2588G>C;5603A>T], p.[Gly863Ala,Gly863del;(Asp1868Ile)]. This patient presents a moderate phenotype with an early onset disease and foveal involvement but with a good preservation of the ff-ERG and peripapillary sparing. As the complex allele c.[2588G>C;5603A>T] has been previously characterized as mild [19], we could hypothesize that c.859-245_859-243delinsTGA could have a severe effect on the phenotype. However, further investigations are needed to clear the role of this variant.

## 3. Discussion

Stargardt disease is a monogenic inherited retinal dystrophy with a relatively high frequency among rare diseases. Nowadays, several therapeutic trials including gene therapy, optogenetics, or oral therapies are ongoing or ready to start [5]. Hence, the importance to identify all the possible candidates is particularly relevant as the genetic confirmation of the clinical diagnosis is always a requirement for the selection of patients. In this context, the screening of *ABCA4* and the identification of the biallelic defects responsible for the disease becomes crucial. In fact, clinical assessment does not always provide enough confidence in the diagnosis of this disease as the phenotype can be very heterogeneous and the presence of phenocopies (diseases with similar phenotype but different genetic backgrounds) is not rare. However, finding the biallelic defects might sometimes be problematic as *ABCA4* is a large gene with more than 1000 pathogenic variants described, including hypomorphic variants and non-canonical splice site variants and in particular, more recently, deep-intronic variants. Coherently to previous literature, in our cohort of STGD1 patients, the screening of the exonic and flanking regions of *ABCA4* led to the recognition of biallelic variants in 68% of the subjects, while the others remained unsolved. In particular, we found only one variant in 70 subjects (13.3%). Among these, 14/70 subjects (20%; 2.6% of the entire cohort) harbored a known pathogenic deep-intronic variant. Previous literature reports a rate of deep-intronic variants among STGD1 patients with only one identified variant ranging from 10 to 50% depending on the methodology used for the screening [10,11,12,22,23]. 

This relatively low performance could be explained by two main hypothesis: (1) as the entire gene was not screened, the second hit may reside in another deep intronic region, the promoter, or UTRs of the gene. In addition, overlooked copy number variants in exonic but also intronic regions may be responsible for the phenotype. To genetically solve these cases, the next step will be to perform genomic sequencing of the *ABCA4* locus. (2) Indeed, as STGD1 has many phenocopies, an exploration of the exome or even genome of these patients might reveal the presence of pathogenic variants on other genes, including genes that have never been associated with inherited retinal dystrophy before. In our screened cohort we found three variants which seem to be recurrent in the European population and have been already reported in different cohorts: c.4253+43G>A on intron 28, c.4539+2064C>T on intron 30 and c.5196+1137G>A on intron 36. Variant c.4253+43G>A was first reported by Zernant et al. [9]. Its pathogenic consequence, leading to a truncating ABCA4 protein, p.[=,Ile1377Hisfs*3], was verified by *in vitro* assays by Sangermano et al. [24]. The authors found this variant always associated with a milder onset phenotype and compound heterozygous with severe variants; hence, they supposed that its effect is mild or hypomorphic. This information is particularly relevant when analyzing family F5207 where, as previously mentioned, CIC05119, unaffected father of the index patient CIC05117, is homozygous for the intronic variant but shows no phenotype. Here we can express two different hypotheses: (1) c.4253+43G>A is indeed hypomorphic and does not cause any disease when homozygous, while it contributes to the disease when *in trans* with c.686T>C which, on the other hand, should be a severe change; (2) variant c.4253+43G>A is mild and CIC05119 will develop the phenotype in the future with a later disease onset associated with foveal sparing. On the other hand, the hypothesis of c.4253+43G>A as a hypomorphic allele does not completely fit with CIC03648 and CIC09817. CIC03648 expresses a particularly severe phenotype, which instead should be alleviated by the presence of a hypomorphic change. However, the presence of another severe deep-intronic variant in *cis* with c.4253+43G>A cannot be excluded. CIC09817, harboring two hypomorphic variants, should not express any phenotype at all (except if they are in *cis* and a third change is present and yet unknown). Ultimately it is possible that the effect of c.4253+43G>A could be variable and might depend on yet unknown individual characteristics. Further investigations are needed to better define the role and effect of this variant in STGD1.Variant c.4539+2064C>T was first reported by Zernant et al. [9] and its effect on the ABCA4 protein, p.[=,Arg1514Leufs*36], was investigated by in vitro assays by Bauwens et al. [11]. Overall, regardless the effect of the second allelic change, the effect of this variant looks severe. This is confirmed in particular in CIC00251 who carries the well-known mild variant c.5882G>A which, in fact, alleviate the overall phenotype.

Finally, variant c.5196+1137G>A was reported, and its effect verified (p.[=,Met1733Glufs*78]) by Braun et al. [22]. Overall, regardless the effect of the second allelic change, the effect of this variant looks mild. However, with the exception of CIC02688 who carries a severe variant, all other patients carry different missense variants, whose effect are difficult to predict. Further investigations would be needed to confirm the role of c.5196+1137G>A in the phenotype. Such a specific genotype-phenotype correlation was never attempted for these deep-intronic variants. Indeed, it is very difficult to have an exhaustive and precise genotype-phenotype correlation in STGD1 [4]. The presence of numerous likely pathogenic missense mutations with unpredictable effects on the function of the protein in vivo complicate their assessment and often the results are assumed from *in silico* predictions, *in vitro* assays or the patients’ phenotype when in compound heterozygosity with well characterized variants (e.g., hypomorphic alleles such as c.5603A>T). Other methods including the analysis of the affected protein domain and the effect(s) of the mutation(s) on the 3D structure of the protein with molecular modeling techniques might be the next step to understand this complex relationship [25,26].

In our study we also investigated potential novel deep-intronic variants that could increase the number of “solved” patients, being aware that the extreme polymorphic nature of *ABCA4* complicates the distinction between real pathogenic variants from benign ones. This issue is particularly relevant in the genetic counseling of the patients who would like to understand the risk of transmission of the disease to the next generation; in fact, the interpretation of the genetic results might be challenging when an unknown variant or a variant of unknown significance is present in the unaffected partner of an affected patient. After the screening of selected deep-intronic regions in our cohort, we found and analyzed 12 variants that were never previously reported as associated with inherited retinal dystrophies and with a MAF ≤ 0.01. This threshold might seem particularly high when looking for variants associated with a recessive disease. However, well known pathogenic variants of *ABCA4* can reach unusual high frequencies. Emblematic is the case of the hypomorphic variant c.5603A>T, which frequency can reach 4.22% in the overall population [19]. Another example is the variant c.5882G>A, with an overall frequency of 0.46% and which can be as high as 1–2% in certain populations [27]. In our study, among all the selected “novel” variants, two showed interesting *in silico* results: c.768+508A>G and c.859-245_859-243delinsTGA. Unfortunately, we were unsuccessful in recontacting the subjects in order to collect the DNA from other relatives to verify the segregation of the variants with the disease, neither to perform a skin biopsy for further functional analysis as performed before [13,22]. According to gnomAD, the variant c.768+508A>G is present in two non-affected individuals at homozygous state, arguing against its pathogenicity. However, this does not exclude a possible hypomorphic effect: as an example, c.4253+43G>A is present in five non-affected subjects in gnomAD, but its pathogenic effects as hypomorphic variants are well proved [24]. It is worth mentioning that we potentially found other interesting novel variants (such as c.859-241A>C, c.1938-703C>T and c.4539+1168C>G). Even though they were not predicted to affect splicing by the in-silico analysis, they might still have potential effects in vivo. In this sense one example is the variant c.4253+43G>A, which is not predicted to cause any effects on splicing by the *in silico* predictions, however, in vitro functional tests demonstrated its potential pathogenicity [24]. The next step will be to test the possible functional effects of these variants on splicing by either minigene approach [14,28] or by extracting RNA from cultured keratinocytes or fibroblasts (taking advantage of the fact that *ABCA4* is expressed at low levels in them [13,15,16,22,24,29,30]), after performing a skin biopsy on the affected patient. However, even these approaches have limitations. In vitro assays could differ from *in vivo* conditions as retina-specific determinants could influence the outcomes of mRNA processing by modulating the non-sense mediated decay processes [31,32]. Furthermore, specifically for minigene approach, it could be difficult to reproduce the natural genomic context in a gene such as *ABCA4* which presents 50 exons and large introns; in this case, the use of larger minigene systems could be more efficient [14]. Other modeling approaches such as induced pluripotent stem cells could be considered as previously successfully done [13,15]; however, cost-effectiveness assessment should be considered. The identification of potential novel pathogenic deep-intronic variants paves the path towards the elaboration of an alternative therapeutic approach consisting in the modulation of *ABCA4* pre-mRNA splicing by using antisense oligonucleotides (AONs). AONs are small molecules which can interfere with splicing by binding their complementary pre-mRNA target. The result could be either the inclusion or the skipping of exons (or pseudoexons), depending on the chosen target [33]. AONs effectiveness have already been successfully tested in vitro in STGD1 [15,16,24,34] and in several other dystrophies [35,36,37,38,39,40,41], and also in vivo [42,43,44], confirming to be a promising therapeutic approach.

## 4. Materials and Methods 

### 4.1. Patients and Preliminary Results

Patients with a presumed diagnosis of STGD1 disease were recruited at the Reference Center for rare diseases, Referet, of the Quinze-Vingts hospital, Paris. Informed consent was obtained from each patient after explanation of the study and its potential outcome. The study protocol adhered to the tenets of the Declaration of Helsinki and was approved by a national ethics committee (CPP Ile de France V, Project number 06693, N◦EUDRACT 2006-A00347-44, 11 December 2006). All the patients and available family members were asked to donate a blood sample for genetic screening for *ABCA4* mutations. There were a total of 1012 blood samples (528 index patients and 484 affected and unaffected family members). DNA samples incorporated in this study were obtained from the NeuroSensCol DNA bank, for research in neuroscience (PI: JA Sahel, co-PI I Audo, partner with CHNO des Quinze-Vingts, Inserm and CNRS). Total genomic DNA was extracted from peripheral whole blood samples by standard salting out procedures according to the manufacturer’s recommendation (Puregen Kit; Qiagen, Courtaboeuf, Les Ulis, France). The first consecutive 211 subjects were screened for known *ABCA4* mutations by microarray analysis on a commercially available microarray (ABCR600, ASPER Biotech, Inc., Tartu, Estonia) [45]. Among them, samples which were excluded for known variants were further investigated for variants in the coding exons and their flanking regions of *ABCA4* by Polymerase Chain Reaction (PCR) and direct Sanger sequencing. The DNA of the other patients included in the cohort was directly Sanger sequenced. At the end of the screening at least two likely pathogenic mutations were identified in 359 index patients (68%), while 169 remained unsolved: 99 (18.7%) with no identified variants and 70 (13.3%) with one single heterozygous mutation. Part of the results of this first genetic screening were previously published [46]. In this study, we further screened the 70 “unsolved” subjects with one heterozygous mutation for known deep-intronic variants on *ABCA4.*

### 4.2. Literature Review

Literature search was performed using Pubmed (https://www.ncbi.nlm.nih.gov/pubmed/), with a last check on January 30th, 2019, in order to collect all reported and validated deep-intronic variants on *ABCA4* in association with STGD1. Additional databases were queried such as The Human Gene Mutation Database (HGMD Professional 2017.4, http://www.hgmd.cf.ac.uk/ac/index.php) last queried on January 30th, 2019 and Leiden Open Variation Database (LOVD V.3.0, https://www.lovd.nl/) last queried on January 30th, 2019. In particular, we included all variants located more than ± 15 pb from the exonic borders, with at least one of the following characteristics: (1) in vitro assays were performed to demonstrate an effect of the variant on the transcript; (2) *in silico* analysis, as well as segregation analysis and MAF were suggestive of a disease causing variant.

The variants included in the study were 24 and are all listed in Table S1.

### 4.3. Genetic Screening

All primers for the intronic mutations were designed according to the following criteria: product obtained by PCR must be between 300 and 600 base pairs (bps); the primer must be 18 to 22 bp in length; the pair of primers must cover at least 50 bp upstream and downstream of the previously reported change. The annealing temperature (TA°C) has to be between 58 °C and 62 °C. The specificity of each primer was then verified by a tool available at the NCBI (National Center Biotechnology Information) website (https://blast.ncbi.nlm.nih.gov/Blast.cgi). We also checked that no reported polymorphism was present in the site of the chosen primers as it would influence the annealing unpredictably. The list of all designed primers is reported in Table S3.

For variants on IVS2 (c.161-23T>G) and IVS28 (c.4253+43G>A), no primers were designed as they were already comprised in the amplicons for exons 3 and 28 respectively. For these variants the sequences previously performed were reviewed. All the investigated intronic regions were amplified in 24 fragments (*ABCA4* RefSeq NM_000350) using oligonucleotides reported in Table S3, a commercially available polymerase (HotFire, Solis Biodyne, Tartu, Estonia), 1.5 mM MgCl_2_ at an annealing temperature of 58 °C for 1 min. The PCR products were enzymatically purified (ExoSAP-IT, USB Corporation, Cleveland, OH, USA, purchased from GE Healthcare, Orsay, France) sequenced and investigated as previously reported [46]. Nucleotide numbering reflects cDNA numbering with +1 corresponding to the A of the ATG translation initiation codon in the reference sequence, according to journal guidelines (www.hgvs.org/ mutnomen). 

Novel variants, distinct from those enlisted in the Table S1, were further investigated. First, the MAF was determined using the Genome Aggregation Database (gnomAD, data available at: https://gnomad.broadinstitute.org/). Only variants with a MAF ≤ 0.01 were included in a more detailed analysis: (1) Evolutionary conservation was investigated using the 46-way Vertebrate Multiz Alignment and Conservation of the University of California Santa Cruz (UCSC, http://genome.ucsc.edu/) genome browser. A nucleotide was considered highly conserved if it was present in all species or was different in just one species among fishes or reptiles, moderately conserved if different in 2 to 5 species (included), and not conserved if different in more than 5 species or in at least one primate (out of 9 included). (2) *In silico* prediction with algorithms capable to predict effects of variants on splicing: SpliceSiteFinder-like (SSF [47]), NNSplice ([48], http://www.fruitfly.org/seq_tools/splice.html), MaxEntScan ([49], http://hollywood.mit.edu/burgelab/maxent/Xmaxentscan_scoreseq.html, Gene Splicer ([50], http://www.cbcb.umd.edu/), ESE finder 3.0 ([51], http://rulai.cshl.edu/tools/ESE). All these algorithms are integrated in the Alamut Visual software (v.2.11-0, Biointeractive Software, France). For all algorithms’ outcomes, changes <10% were considered as having no effects; changes between 10% and 30% were considered mild; changes between 30% and 60% were considered moderate; changes >60% were considered strong. 

### 4.4. Phenotypic Analysis

A phenotypic analysis was performed on patients whose screening revealed the presence of a deep-intronic variant. Clinical charts were reviewed, and the following data collected: demographic data (i.e., date of birth, ethnicity, family history, smoking history); age of onset (defined as the age when symptoms related to the disease first occurred); symptoms at onset; age at the last visit and duration of the disease. When possible, the results of the clinical examination were also included from the last most comprehensive visit available: best corrected visual acuity (BCVA) with the Early Treatment Diabetic Retinopathy Study (ETDRS) chart, kinetic and static perimetry, and color vision with the desaturated Farnsworth Panel D-15, full-field and multifocal electroretinography (ff-ERG and mf-ERG; Espion E2; for full field ERG; Diagnosys, Lowell, MA, USA; and Veris II for multifocal ERG; EDI, Redwood City, CA, USA), color fundus photograph (FP; Topcon, Tokyo, Japan), short-wavelength fundus autofluorescence (SW-AF), near-infrared fundus autofluorescence (NIR-AF), spectral domain optical coherence tomography (OCT; Heidelberg retina angiograph [HRA] II or Spectralis HRA+OCT; Heidelberg Engineering, Dossenheim, Germany). Patients were classified for FP, AF, ff-ERGs and OCT according to the criteria summarized in Table S4. Briefly, color fundus photographs were classified in 4 stages according to the focal or diffuse involvement of the posterior pole with flecks and chorioretinal atrophy (Figure S6) [52]. Autofluorescence images were stratified in 5 groups: group 1: central lesion with jagged borders, group 2: central lesion with extensive fundus changes; group 3: central lesion with smooth borders and an hyperautofluorescent ring-like halo in SW-AF and NIR-AF; group 4: central lesion with smooth borders and no hyperautofluorescent NIR-AF ring; group 5: small discrete central lesion better visualized in NIR-AF (Figures S7 and S8) [53]. A single horizontal high-resolution OCT B-Scan was used to evaluate the preservation of the ellipsoid zone (EZ) and RPE through the fovea to evaluate the presence or absence of foveal sparing (Figure S9) [54]. Finally, all patients underwent electrophysiological assessment, including ff-ERG and mf-ERG, incorporating the minimum standards of the International Society for Clinical of Electrophysiology of Vision (ISCEV) [55,56]. The patient data set was compared against those of 30 healthy subjects (15 younger than 30 years old and 15 older; Table S5). The limits of ERG normality were defined for all the components of the ERG as the mean value ± 2 standard deviations. All the components of the ERG from each eye were taken into account when classifying patients into the three ERG groups defined by Lois et al. [57]: Group 1 has abnormal mf-ERG with normal ff-ERG; in Group 2 there were mf-ERG abnormalities with abnormal amplitudes and implicit times in response to all the light adapted stimulations (cone dysfunction); Group 3 has additional rod dysfunction (i.e., abnormal amplitudes and implicit times to all stimulations). The overall classification was based on the more severe eye when the ERG group was different between eyes in the same patient.

## 5. Conclusions

Overall, our screening allowed for the recognition of the second mutated allele in 14 subjects (plus 2 subjects with novel variants that need further investigations) among 70 patients with only one pathogenic variant in the exonic part of *ABCA4.* We also provided a comprehensive genotype-phenotype correlation for the three recurrent deep-intronic variants identified in the cohort. These results are relevant as they might help clinicians in patient counseling and prognostic predictions. Furthermore, they may help with the identification of fast or slow disease progressers, which constitutes crucial information to select and monitor subjects in clinical therapeutic trials. 

## Figures and Tables

**Table 1:** Complete genotype and segregation analysis (when available) of patients carrying a deep-intronic variant on *ABCA4*. Nucleotide positions and translation correspond to CCDS747.1 and NP_000341.2, respectively.

			Allele 1			Allele 2		
Patient ID	Family ID		Exon/Intron	Nucleotide Change	Protein Change	Exon/Intron	Nucleotide Change	Protein Change
CIC00251	174	Index	42	c.5882G>A	p.(Gly1961Glu)	IVS30	c.4539+2064C>T	p.[=,Arg1514Leufs*36]
CIC00454	174	Unaffected father	42	c.5882G>A	p.(Gly1961Glu)	reference sequence		
CIC00455	174	Unaffected mother	reference sequence			IVS30	c.4539+2064C>T	p.[=,Arg1514Leufs*36]
CIC01275	765	Index	10	c.1344del	p.(Ile449Metfs*3)	IVS30	c.4539+2064C>T	p.[=Arg1514Leufs*36]
CIC01276	765	Unaffected mother	reference sequence			IVS30	c.4539+2064C>T	p.[=Arg1514Leufs*36]
CIC01277	765	Unaffected father	10	c.1344del	p.(Ile449Metfs*3)	reference sequence		
CIC02688	960	Index	42	c.5888del	p.(Pro1963Argfs*11)	IVS36	c.5196+1137G>A	p.[=,Met1733Glufs*78]
CIC03648	1602	Index	IVS38	c.5461-10T>C	p.[Thr1821Aspfs*6, Thr1821Valfs*13]	IVS28	c.4253+43G>A	p.[=,Ile1377Hisfs*3]
			40	c.5603A>T	p.(Asn1868Ile)			
CIC03649	1602	Unaffected aunt	IVS38	c.5461-10T>C	p.[Thr1821Asp*6, Thr1821Valfs*13]	reference sequence		
			40	c.5603A>T	p.(Asn1868Ile)			
CIC04422	2138	Index	28	c.4139C>T	p.(Pro1380Leu)	IVS36	c.5196+1137G>A	p.[=,Met1733Glufs*78]
CIC04795	2391	Index	28	c.4139C>T	p.(Pro1380Leu)	IVS36	c.5196+1137G>A	p.[=,Met1733Glufs*78]
CIC06528	3493	Index	IVS40	c.5714+5G>A	p.[=,Glu1863Leufs*33]	IVS30	c.4539+2064C>T	p.[=,Arg1514Leufs*36]
CIC07955	3493	Affected cousin	IVS40	c.5714+5G>A	p.[=,Glu1863Leufs*33]	IVS30	c.4539+2064C>T	p.[=,Arg1514Leufs*36]
CIC06981	3831	Index	22	c.3322C>T	p.(Arg1108Cys)	IVS36	c.5196+1137G>A	p.[=,Met1733Glufs*78]
CIC08281	4645	Index	43	c.5914G>A	p.(Gly1972Arg)	IVS28	c.4253+43G>A	p.[=,Ile1377Hisfs*3]
CIC07459	4128	Index	13	c.1804C>T	p.(Arg602Trp)	IVS36	c.5196+1137G>A	p.[=,Met1733Glufs*78]
CIC07460	4128	Unaffected mother	reference sequence			IVS36	c.5196+1137G>A	p.[=,Met1733Glufs*78]
CIC07461	4128	Unaffected father	13	c.1804C>T	p.(Arg602Trp)	reference sequence		
CIC08968	4128	Unaffected sister	reference sequence			reference sequence		
CIC08809	5010	Index	6	c.688T>G	p.(Cys230Gly)	IVS30	c.4539+2064C>T	p.[=,Arg1514Leufs*36]
CIC09117	5207	Index	6	c.686T>C	p.(Leu229Pro)	IVS28	c.4253+43G>A	p.[=,Ile1377Hisfs*3]
CIC09118	5207	Unaffected mother	6	c.686T>C	p.(Leu229Pro)	reference sequence		
CIC09119	5207	Unaffected father	IVS28	c.4253+43G>A	p.[=,Ile1377Hisfs*3]	IVS28	c.4253+43G>A	p.[=,Ile1377Hisfs*3]
CIC09817	5639	Index	40	c.5603A>T	p.(Asn1868Ile)	IVS28	c.4253+43G>A	p.[=,Ile1377Hisfs*3]
CIC10544	6093	Index	13	c.1804C>T	p.(Arg602Trp)	IVS36	c.5196+1137G>A	p.[=,Met1733Glufs*78]

**Table 2:** In-silico analysis and conservation study of variants found during our screening, which were never associated with Stargardt disease and with a minor allele frequency (MAF) ≤ 0.01. MAF data were obtained from the gnomAD database (https://gnomad.broadinstitute.org/). In bold, the two novel variants with moderate to strong predicted changes by the analysis. MAF: Minor allele frequency, SNP: single nucleotide polymorphism.

# Subject	Location	Variant (CCDS747.1)	SNP ID	Nucleotide Conservation	MAF (Allele Count/Allele Total/Number of Homozygous)	SSF	MaxEntScan	NNSplice	GeneSplicer	ESE Finder
CIC03520	IVS6	c.768+353T>C	rs79372932	Not conserved	0.003281 (103/31392/0)	No changes	No changes	Mild activation of an acceptor site	Mild activation of an acceptor site	No changes
**CIC03956**	**IVS6**	**c.768+508A>G**	**rs77933221**	**Moderately Conserved**	**0.007579 (238/31404/2)**	**Strong inactivation of a donor site and activation of an acceptor site**	**Moderate inactivation of a donor site and activation of an acceptor site**	**No changes**	**No changes**	**Formations of binding sites for SF2/ASF**
CIC08792	IVS6	c.769-775C>T	-	Not conserved	Absent	No changes	No changes	No changes	No changes	No changes
CIC03956	IVS7	c.859-364C>T	rs544917926	Not conserved	0.003154 (99/31388/2)	No changes	No changes	No changes	No changes	No changes
CIC01916	IVS7	c.859-256A>G	rs538160992	Not Conserved	0.00009553 (3/31404/0)	No changes	No changes	No changes	No changes	No changes
**CIC01916**	**IVS7**	**c.859-245_859-243delinsTGA**	-	**C and T highly conserved, A not conserved**	**Absent**	**Strong activation of a donor site**	**Strong activation of a donor site**	**Strong activation of a donor site**	**Strong activation of a donor site**	**Formations of binding sites for SF2/ASF and Srp40**
CIC10548	IVS7	c.859-241A>C	rs56378813	Highly conserved	0.0009871 (31/31404/0)	No changes	No changes	No changes	No changes	No changes
CIC01916	IVS7	c.859-235T>C	-	Not Conserved	Absent	No changes	No changes	No changes	No changes	No changes
CIC00952 CIC00973 CIC01413 CIC02688 CIC09897 CIC10529 CIC10548 CIC10577	IVS13	c.1938-703C>T	-	Highly conserved	Absent	No changes	No changes	No changes	No changes	No changes
CIC06173	IVS14	c.2160+462A>C	-	Not conserved	Absent	No changes	No changes	No changes	No changes	No changes
CIC09897	IVS30	c.4539+1168C>G	-	Moderately Conserved	Absent	No changes	No changes	No changes	No changes	Formations of binding sites for SF2/ASF
CIC06353	IVS44	c.6148-489C>T	rs894440427	Not conserved	0.0003821 (12/31408/0)	No changes	No changes	No changes	Mild inactivation of donor site and activation of acceptor site	No changes

**Table 3:** Retrospective data collection of the phenotype of the subject harboring a deep-intronic variant in *ABCA*. Aoo: Age of onset; Aoe: Age at the time of examination; BCVA: best corrected visual acuity: RE: right eye; LE: left eye; AF: autofluorescence; OCT: optical coherence tomography; ERG: electroretinogram; RPE: Retinal pigment epithelium.

Patient CIC#	Aoo (years)	Aoe (years)	Duration (years)	Sex	Active Smoking	History	Symptoms at Onset	BCVA RE/LE	Color Vision (axis)	Binocular Perimetry	Fundus grading	AF				
												Group	Atrophy RPE	Peripapillary Sparing	SD-OCT Foveal Sparing	ERG
00251	10	44	34	M	No	Myopic	Decreased VA	20/200 / 20/200	Normal	Central scotoma 20°	III	II	Present	Atrophy	No	I
01275	11	16	5	M	No	Myopic	Decreased VA photophobia	20/200 / 20/250	Deutan	Central scotoma 20°	II	II	Present	Flecks	No	III
02688	-	63	-	F	-	-	-	-	-	-	III	II	Present	Yes	Yes	II
03648	16	26	10	F	No	Negative	Decreased VA	20/160 / 20/160	Normal	Central scotoma 5°	III	II	Present	Flecks	No	II
10544	-	49	-	F	-	Negative	-	20/32 / 20/25	Normal	-	II	II	Present	Yes	Yes	I
04795	-	52	-	F	-	-	Decreased VA photophobia	20/500 / 20/200	Multiple	Central scotoma 20°	III	II	Present	Atrophy	Yes	II
06528	26	56	30	M	No	Negative	Decreased VA	20/500 / 20/500	Deutan	Central scotoma 50°	IV	II	Present	Atrophy	No	III
06981	61	72	11	M	No	Negative	Decreased VA	20/50 / 20/50	Normal	Paracentral scotoma 30°	IV	II	Present	Flecks	Yes	I
08281	25	45	20	F	No	Right eye amblyopic	Decreased VA	20/125 / 20/125	Normal	Central scotoma 10°	I	IV	Present	Yes	No	I
07459	15	22	7	F	No	Negative	Decreased VA	20/200 / 20/160	Tetartan	Central scotoma 20°	II	II	Absent	Flecks	No	II
08809	14	38	24	F	No	Negative	Decreased VA	20/320 / 20/250	Protan	Central scotoma 30°	IV	II	Present	Yes	No	II
09117	41	47	6	F	No	Negative	Decreased VA photophobia	20/50 / 20/200	Deutan	Paracentral scotoma 10°	I	II	Present	Yes	Yes	I
09817	50	66	16	F	Yes	Negative	Decreased VA	20/160 / 20/32	Protan Tritan	Paracentral scotoma 40°	III	II	Present	Yes	Yes	II
04422	56	72	16	M	Past	Negative	Decreased VA night blindness	20/50 / 20/32	Tritan	Paracentral scotoma 20°	IV	II	Present	Yes	Yes	III
01916	21	40	19	M	No	Negative	Decreased VA	20/500 / 20/200	Normal	Central scotoma 30°	IV	II	Present	Yes	No	I
03956	-	-	-	F	-	-	-	-	-	-	-	-	-	-	-	-

## Supplementary Materials

Supplementary materials can be found at https://www.mdpi.com/1422-0067/20/20/5053/s1.

## Abbreviations

ABCA4	ATP-binding cassette transporter 4
AONs	antisense oligonucleotides
BCVA	best corrected visual acuity
bp	base pair
ECD	Extracellular domain
ERG	Electroretinogram
ETDRS	Early Treatment Diabetic Retinopathy Study
EZ	ellipsoid zone
ff-ERG	Full field electroretinogram
FP	Fundus Photograph
ISCEV	International Society for Clinical of Electrophysiology of Vision
MAF	minor allele frequency
mf-ERG	Full field electroretinogram
NBD	nucleotide binding domain
NIR-AF	Near infrared fundus autofluorescence
OCT	optical coherence tomography
RPE	Retinal pigment epithelium
STGD1	Autosomal recessive Stargardt disease
SW-AF	Short wavelength fundus autofluorescence
