# Supplementary material for: Prevalence of ABCA4 Deep-Intronic Variants and Related Phenotype in An Unsolved “One-Hit” Cohort with Stargardt Disease
Source: Int J Mol Sci. 2019 Oct 11;20(20):5053. doi: 10.3390/ijms20205053 (PMC6829239; doi:10.3390/ijms20205053)
Supplement: Supplementary file 1 [file ijms-20-05053-s001.pdf]

Table S1. List of all variants screened in this study.

Genome starting position (hg19)	Location on *ABCA4*	Variant (NM_000350.2)	Effect on protein (*in vitro* assays, when available) (NP_000341.2)	Reference
g.94577158	IVS2	c.161-23T>G	p.[=,Cys54Serfs*14]	Bauwens 2019 [1]
g.94566773	IVS5	c.570+1798A>G	-	Zernant 2014 [2]
g.94563992	IVS6	c.768+358C>T	-	Zernant 2014 [2]
g.94549781	IVS6	c.769-784C>T	p.[=,Leu257Asp*3]	Sangermano 2019 [3]
g.94546814	IVS7	c.859-540C>G	p.(Phe287Tyrfs*33)	Bauwens 2019 [1]
g.94546780	IVS7	c.859-506G>C	p.[Phe287Thrfs*32,=]	Sangermano 2019 [3]
g.94526934	IVS13	c.1938-619A>G	-	Zernant 2014 [2]
g.94525509	IVS14	c.2160+584A>G	-	Zernant 2014 [2]
g.94509799	IVS20	c.3050+370C>T	-	Zernant 2014 [2]
g.94496509	IVS28	c.4253+43G>A	p.[=, Ile1377Hisfs*3]	Sangermano 2019 [3]
g.94493901	IVS30	c.4539+1100A>G	p.[Arg1514Valfs*31,Arg1514Glyfs*3,=]	Sangermano 2019 [3]
g.94493895	IVS30	c.4539+1106C>T	p.[Arg1514Glyfs*3, Arg1514Valfs*31]	Bauwens 2019 [1]
g.94493272	IVS30	c.4539+1729G>T	-	Zernant 2014 [2]
g.94493000	IVS30	c.4539+2001G>A	p.[=,Arg1514Leufs*36]	Albert 2018 [4]
g.94492973	IVS30	c.4539+2028C>T	p.[=,Arg1514Leufs*36]	Albert 2018 [4]
g.94492937	IVS30	c.4539+2064C>T	p.[=,Arg1514Leufs*36]	Bauwens 2019 [1]
g.94484039	IVS36	c.5196+1013A>G	-	Schulz 2017 [5]
g.94484082	IVS36	c.5196+1056A>G	-	Braun 2013 [6]
g.94484000	IVS36	c.5196+1136C>A	-	Bauwens 2015 [7]
g.94484001	IVS36	c.5196+1137G>A	p.[=,Met1733Glufs*78]	Braun 2013 [6]
g.94483865	IVS36	c.5196+1159G>A	-	Bauwens 2015 [7]
g.94483922	IVS36	c.5196+1216C>A	-	Braun 2013 [6]
g.94481967	IVS36	c.5197-557G>T	p.(Met1733*)	Bauwens 2019 [1]
g.94468019	IVS44	c.6148-471C>T	-	Zernant 2014 [2]

Table S2. Evolutionary conservation of the two novel deep intronic variants with predicted *in silico* effect on splicing. Nucleotidic genomic and complementary DNA (cDNA) positions correspond to GRCh37 (hg19) and CCDS747.1, respectively.

Nucleotide position (cDNA)	c.768+508	c.859-245_859-243
**Nucleotide position (genomic)**	**g.94563842T**	**g.94546517_94546519ATG**
**Altered residue**	**C**	**TCA**
Human	AACCATTGAGG	GGCTCATGCCTGT
Chimp	AACCATTGAGG	GGCTCATGCCTGT
Gorilla	AACCATTGAGG	GGCTCATGCCTGT
Orangutan	AACTATTGAGG	GGCTCACGCCTGT
Rhesus	AACTATTGAGG	GGTTCATGCCTGT
Baboon	AACTATTGAGG	GGTTCATGCCTGT
Marmoset	AACTTTTGAGG	----------------
Tarsier	AAGTTTTGGGG	----------------
Mouse_lemur	AACATTTGACA	----------------
Bushbaby	AGCCTTTGAGG	----------------
Tree_shrew	AAGCTCTGAGG	----------------
Mouse	ACCTTTGGGGA	----------------
Rat	ACC--TGGGGA	----------------
Kangaroo_rat	AACTTTTGGGG	----------------
Guinea_pig	AACTTTTGAGG	----------------
Squirrel	CATTGTGTGGG	----------------

Rabbit	AACATTTGGGG	----------------
Pika	AACATTTGGGG	----------------
Alpaca	----------------	----------------
Dolphin	AAACTTTGGGG	-------ACCTGC
Cow	AAGCTCTGAGG	----------------
Horse	AACCTTTGGGG	----------------
Cat	AACCTTTGTGG	----------------
Dog	AACCTTTGTGG	----------------
Microbat	AACGTTTGGGG	----------------
Megabat	AAACTTTGCGG	----------------
Hedgehog	----------------	----------------
Shrew	AAGCTTTAGAG	----------------
Elephant	AAC-TTGTTGC	----------------
Rock_hyrax	AAC-TTACTAG	----------------
Tenrec	---------------	----------------
Armadillo	ATC-CTCGGGA	----------------
Sloth	AACATTTGGAA	----------------

Table S3. List of primers designed to investigate the deep intronic variants included in this study.

Primer ID	Forward primer (FASTA format)	Primer ID	Reverse primer (FASTA format)	Amplicon size (base pair)
ABCA4_int5F	CCGAAACTAGACAAGGGGAAC	ABCA4_int5R	AAAGCATCCTGGGAAGTGGG	508
ABCA4_int6F	TGGCATGTTTGTGTCCACTCT	ABCA4_int6R	CAGGTCCCAAGCAGTCTGTC	413
ABCA4_int6bisF	GATACTTGGATGAGAATTAC	ABCA4_int6bisR	AGCTCCAGAGACTGATGTGA	258
ABCA4_int7F	CCCAAGAACTGGCTTAACAG	ABCA4_int7R	GCTCCAACGTTTGGTTTGAC	591
ABCA4_int13F	ATGCTGGAATTGGGCCCTTT	ABCA4_int13R	AGGACTGACAAGGGCAAGTG	388
ABCA4_int14F	CAGTAGCAGTAGGGGAGGAGA	ABCA4_int14R	AAAATGAAGGATAGCAGCGCA	278
ABCA4_int20F	GAGCAGCTGATCGATCCCC	ABCA4_int20R	TCCCTTTTCCTCCCTCCTGT	354
ABCA4_int30F	CCTCAGCCTCATCAGCCAAT	ABCA4_int30R	GCGTGGAAGTAAGGGTTCGT	603
ABCA4_int30bisF	ATACATGCACAGCCAGCATC	ABCA4_int30bisR	CATACCTGTGCTTTCCCACT	274
ABCA4_int36F	TGGAGACCAACACAAATGACC	ABCA4_int36R	GCCAGCCCCAAGTGTGTAAA	392
ABCA4_int36bisF	CAACTCATTTATCTACCGGAC	ABCA4_int36bisR	TTCTTGGCTTCACAAAGCTCA	381
ABCA4_int44F	TAGATCCCCTCCTGCGCAT	ABCA4_int44R	TCCCAGACCTGTTGATCCCA	261

Table S4. Clinical criteria used to describe patients' phenotype in this study. FP= Fundus photograph; SW-AF= Short-Wavelength Fundus Autofluorescence; NIR-AF= Near Infrared Fundus Autofluorescence; ERG= Electroretinogram; OCT= Optical coherence tomography; RPE: Retinal pigment epithelium; EZ: Ellipsoid zone.

	Group/Stage	Criteria	Reference
**Age of onset**	n.a.	Age when symptoms first occurred.	Lois N et al. 2001 [8]
**FP** (Supplementary Fig 1)	Stage 1	Central macular atrophy with foveal or perifoveal flecks.	Fishman GA et al. 1976 [9]
	Stage 2	Previous stage + numerous flecks, extending beyond the vascular arcades or the optic disc.	
	Stage 3	Macular chorioretinal atrophy and resorption of flecks.	
	Stage 4	Extensive chorioretinal atrophy.	
**SW-AF and NIR-AF** (Supplementary Fig 2 and 3)	Group 1	Central lesion with irregular border.	Duncker T, et al 2014 [10]
	Group 2	Extensive fundus changes.	
	Group 3	Central lesion with smooth border and hyperautofluorescent ring in both SW-AF e NIR-AF.	
	Group 4	Central lesion with smooth border and hyperautofluorescent ring only in SW-AF.	
	Group 5	Small central lesion, better detectable in NIR-AF.	
	Peripapillary sparing	Absence of any alterations within 0.6 mm of eccentricity from the optic disc.	Cideciyan AV et al 2005 [11]
	Flecks in the peripapillary area	Presence of flecks within 0.6 mm of eccentricity from the optic disc.	
	Peripapillary area involved by atrophy	Atrophy within 0.6 mm of eccentricity from the optic disc.	
**ERG**	I	All components of ERG are within normal ranges	Lois N et al. 2001 [8]
	II	Abnormal amplitudes and implicit times in response to all the light adapted stimulations	
	III	Abnormal amplitudes and implicit times in response to all stimulations	
**SW-FAF and OCT** (Supplementary Fig 4)	Speared Fovea	Foveal sparing with late onset, intact RPE and EZ on OCT	Fujinami K et al. 2013 [12]
	Involved Fovea	Foveal EZ and/or RPE atrophy and early age of onset	

Table S5. Electrophysiological data in a group of 30 healthy volunteers. ERG: electroretinogram; SD: Standard deviation; IT: implicit time; Amp: amplitude.

	Dark Adapted 0.01 ERG		Dark-Adapted 10.0 ERG				Light Adapted 3.0 Flicker		Light Adapted 3.0 ERG			
**Age Group**	**b-wave IT, ms**	**b-wave Amp, μV**	**a-wave IT, ms**	**a-wave Amp, μV**	**b-wave IT, ms**	**b-wave Amp, μV**	**Period, ms**	**Amp, μV**	**a-wave IT, ms**	**a-wave Amp, μV**	**b-wave IT, ms**	**b-wave Amp, μV**
13-30 y (n=15)	80-105	176-447	8.5-13	254-498	28-53	270-635	24-28	78-220	12-15	32-70	27-32	106-305
Mean (SD)	93.7 (4.9)	301.2 (75.2)	11 (1.5)	391 (63.5)	45 (3.9)	487 (98.25)	25.1 (0.7)	140 (27.25)	13.4 (0.6)	57.1 (10.5)	28.5 (1.1)	201 (42.58)
19.46 ± 6.41												
31-65 y (n=15)	81-107	101-389	09-13	198-501	30-55	300-657	24-29	67-214	12-15	21-65	26-32	92-235
Mean (SD)	94.23 (6.2)	251 (72.2)	12 (1.5)	322.1 (100.5)	49.54 (5.1)	442 (88.25)	25 (1.1)	99.25 (21.46)	13.5 (1)	40.74 (12.75)	28.21 (0.7)	157 (40.5)
47.26 ± 9.76												

.

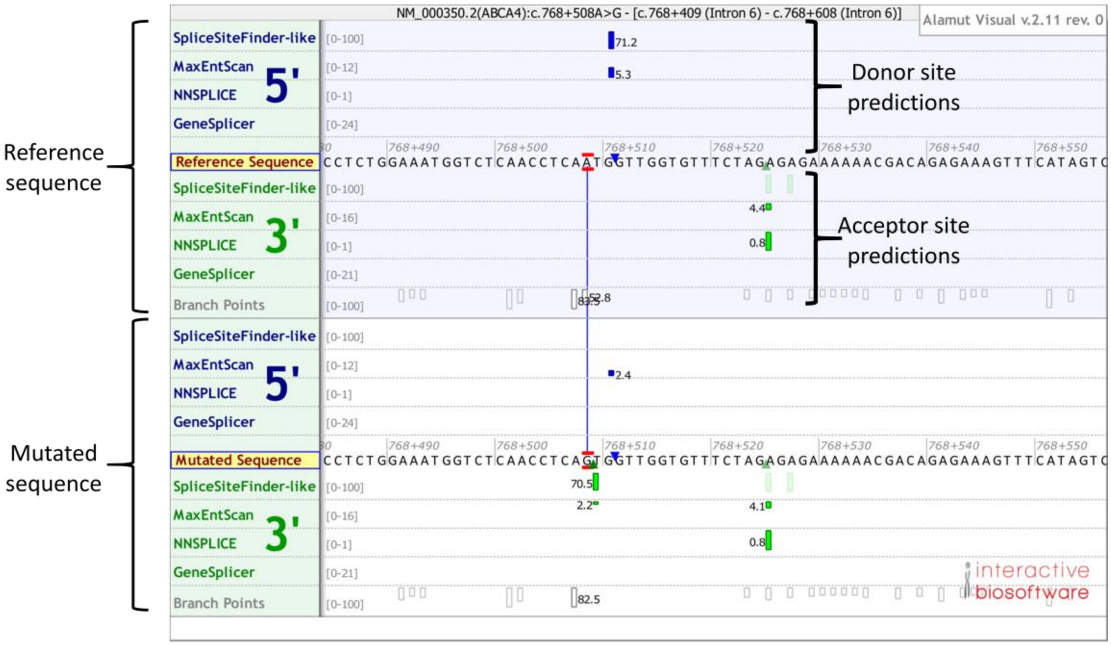

Figure S1. *In silico* predictions for variant c.768+508A>G performed on Alamut Visual v.2.11. While NNSPLICE and GeneSplicer report no changes between the reference and the mutated sequences, SSFinder and MaxEntScan predict the activation of an acceptor site and a deactivation of a donor site at the level of the variant.

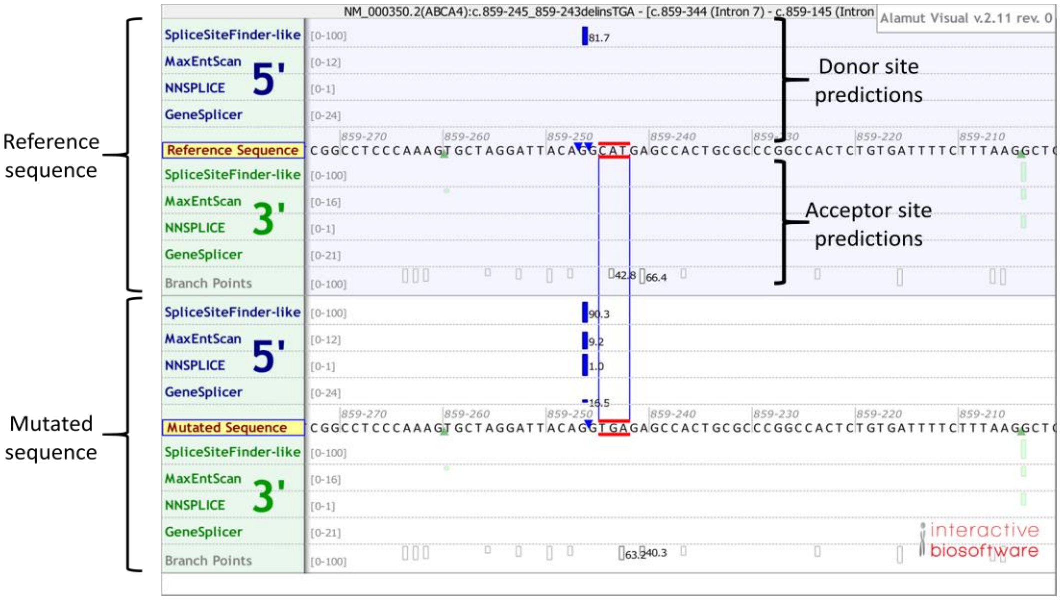

Figure S2. *In silico* predictions for variant c.859-245_859-243delinsTGA performed on Alamut Visual v.2.11. All algorithms used predicted a strong activation of a donor site in correspondence of the variant.

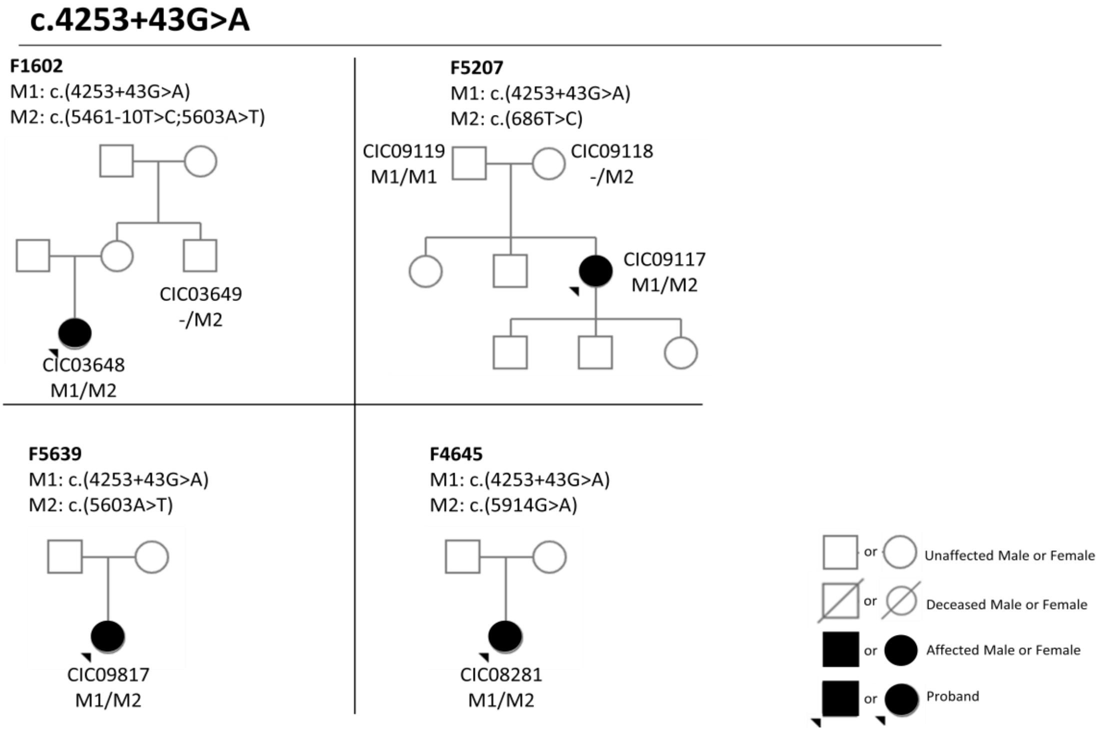

Figure S3. Pedigrees of the four families where the mutation c.4253+43G>A is carried by the respective probands.

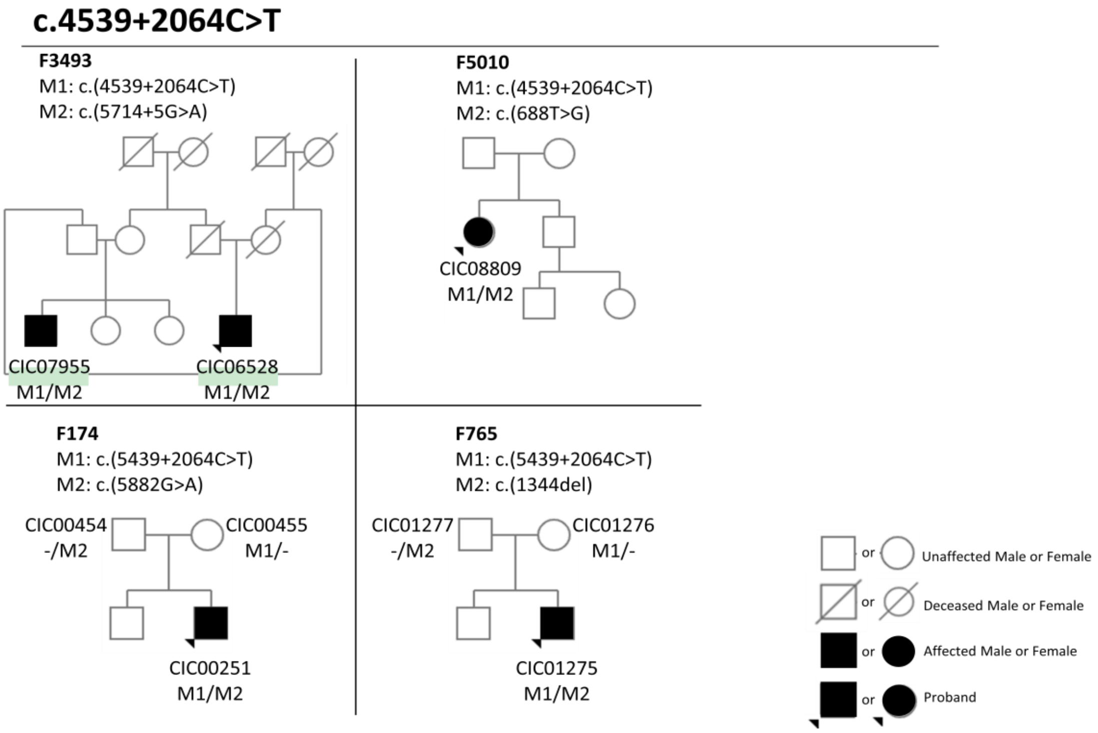

Figure S4. Pedigrees of the four families where the mutation c.4539+2064C>T is carried by the respective probands.

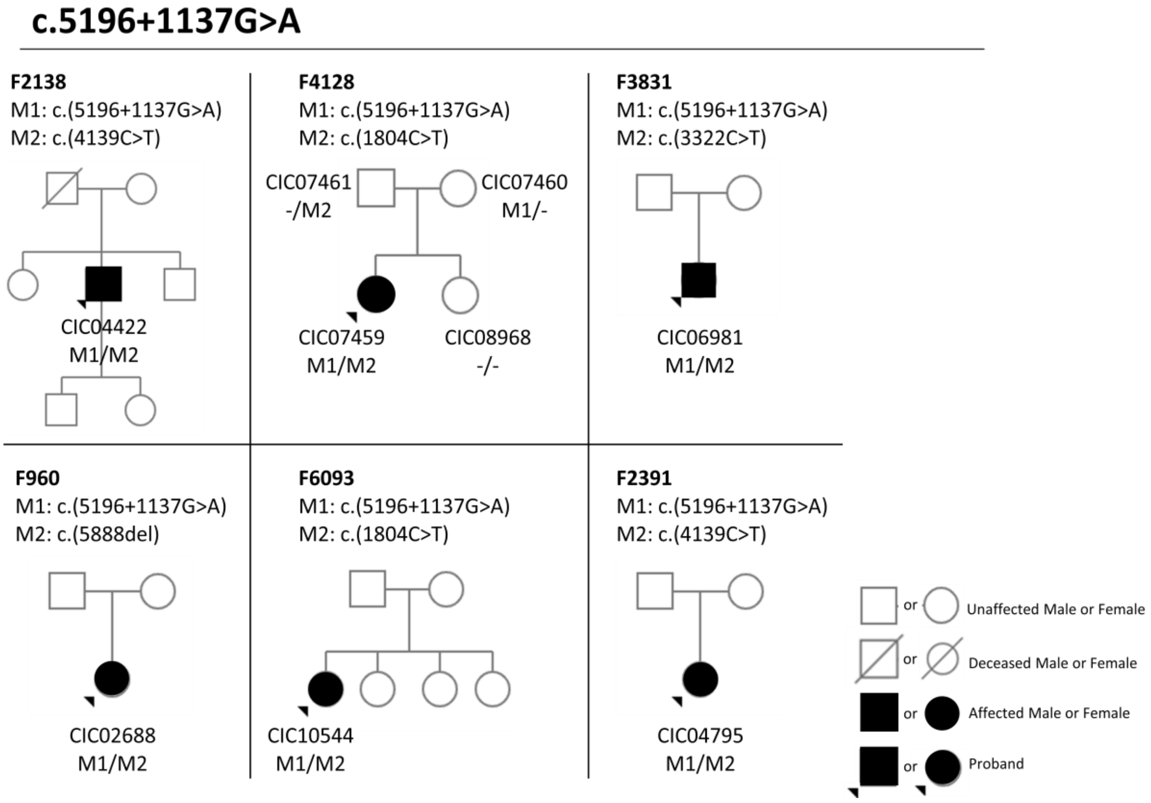

Figure S5. Pedigrees of the six families where the mutation c.5196+1137G>A is carried by the respective probands.

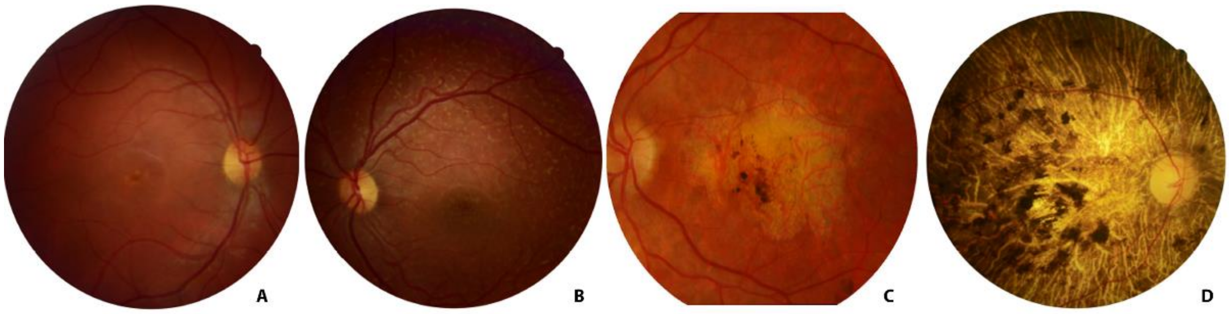

Figure S6. Clinical stages of Stargardt disease in color fundus photographs: stage 1 (A); stage 2 (B); stage 3 (C); stage 4 (D).

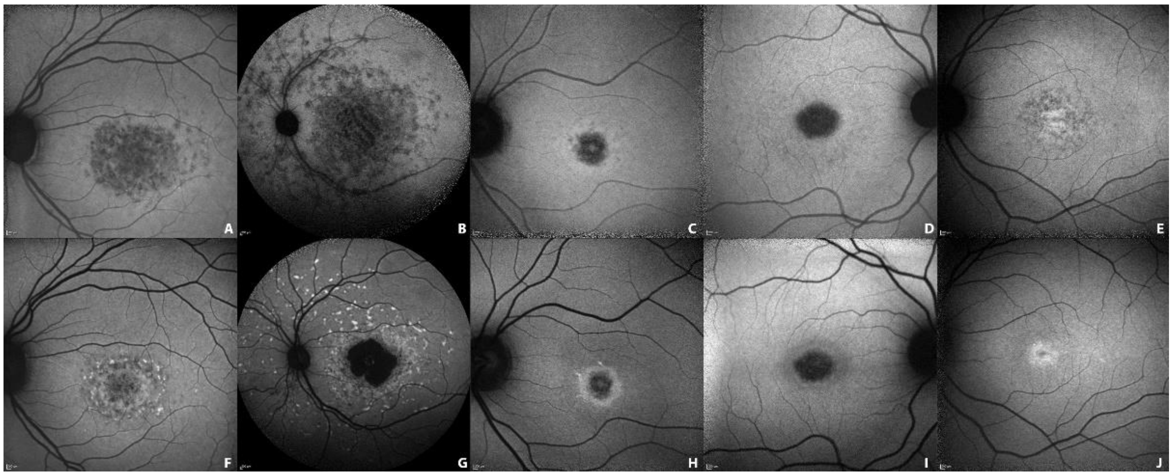

Figure S7. Classification of patients using near-infrared (A-E) and short-wavelength (F-J) fundus autofluorescence techniques. Group 1 (A and F); group 2 (B and G); group 3 (C and H); group 4 (D and I); group 5 (E and J).

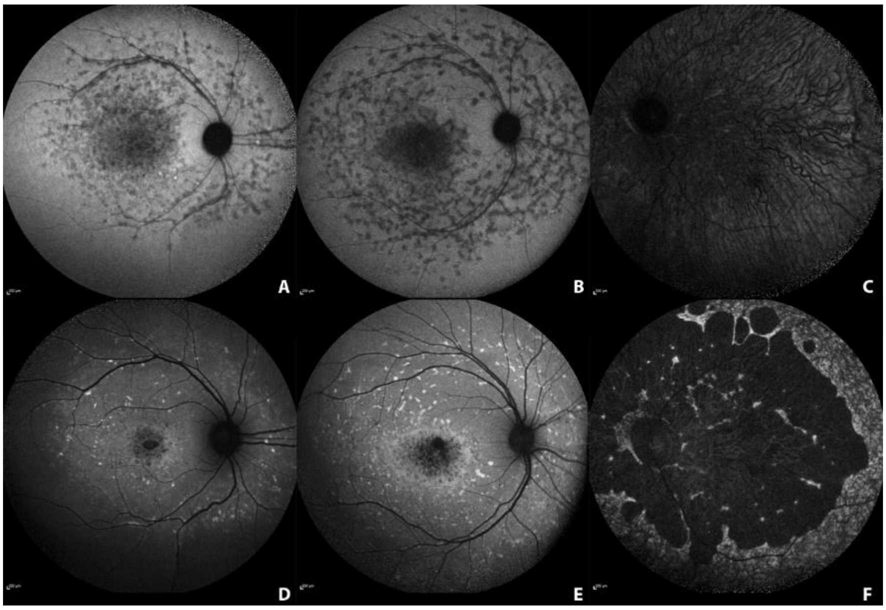

Figure S8. Peripapillary sparing assessed by near-infrared (A-C) and short-wavelength (D-F) fundus autofluorescence. Peripapillary area spared (A and D); flecks in the peripapillary area (B and E); atrophy in the peripapillary area (C and F).

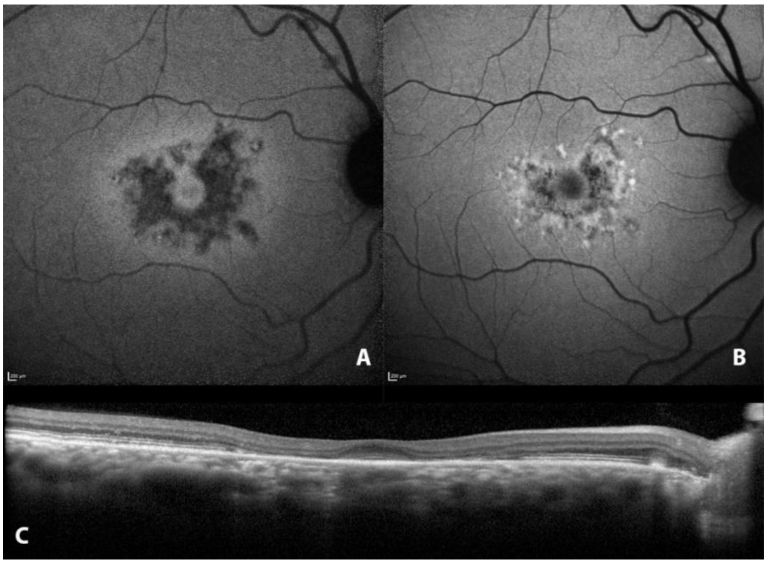

Figure S9. Foveal sparing as assessed by near-infrared (A) and short-wavelength (B) fundus autofluorescence and spectral-domain optical coherence tomography (C).

References

1. Bauwens, M.; Garanto, A.; Sangermano, R.; Naessens, S.; Weisschuh, N.; Zaeytijd, J.D.; Khan, M.; Sadler, F.; Balikova, I.; Cauwenbergh, C.V.; et al. ABCA4 -associated disease as a model for missing heritability in autosomal recessive disorders: novel noncoding splice, cis -regulatory, structural, and recurrent hypomorphic variants. *Genetics in Medicine* **2019**, 1.

2. Zernant, J.; Xie, Y.A.; Ayuso, C.; Riveiro-Alvarez, R.; Lopez-Martinez, M.-A.; Simonelli, F.; Testa, F.; Gorin, M.B.; Strom, S.P.; Bertelsen, M.; et al. Analysis of the ABCA4 genomic locus in Stargardt disease. *Hum. Mol. Genet.* **2014**, *23*, 6797–6806.
3. Sangermano, R.; Garanto, A.; Khan, M.; Runhart, E.H.; Bauwens, M.; Bax, N.M.; van den Born, L.I.; Khan, M.I.; Cornelis, S.S.; Verheij, J.B.G.M.; et al. Deep-intronic ABCA4 variants explain missing heritability in Stargardt disease and allow correction of splice defects by antisense oligonucleotides. *Genet. Med.* **2019**.
4. Albert, S.; Garanto, A.; Sangermano, R.; Khan, M.; Bax, N.M.; Hoyng, C.B.; Zernant, J.; Lee, W.; Allikmets, R.; Collin, R.W.J.; et al. Identification and Rescue of Splice Defects Caused by Two Neighboring Deep-Intronic ABCA4 Mutations Underlying Stargardt Disease. *Am. J. Hum. Genet.* **2018**, *102*, 517–527.
5. Schulz, H.L.; Grassmann, F.; Kellner, U.; Spital, G.; Rüther, K.; Jägle, H.; Hufendiek, K.; Rating, P.; Huchzermeyer, C.; Baier, M.J.; et al. Mutation Spectrum of the ABCA4 Gene in 335 Stargardt Disease Patients From a Multicenter German Cohort-Impact of Selected Deep Intronic Variants and Common SNPs. *Invest. Ophthalmol. Vis. Sci.* **2017**, *58*, 394–403.
6. Braun, T.A.; Mullins, R.F.; Wagner, A.H.; Andorf, J.L.; Johnston, R.M.; Bakall, B.B.; Deluca, A.P.; Fishman, G.A.; Lam, B.L.; Weleber, R.G.; et al. Non-exomic and synonymous variants in ABCA4 are an important cause of Stargardt disease. *Hum. Mol. Genet.* **2013**, *22*, 5136–5145.
7. Bauwens, M.; De Zaeytijd, J.; Weisschuh, N.; Kohl, S.; Meire, F.; Dahan, K.; Depasse, F.; De Jaegere, S.; De Ravel, T.; De Rademaeker, M.; et al. An augmented ABCA4 screen targeting noncoding regions reveals a deep intronic founder variant in Belgian Stargardt patients. *Hum. Mutat.* **2015**, *36*, 39–42.
8. Lois, N.; Holder, G.E.; Bunce, C.; Fitzke, F.W.; Bird, A.C. Phenotypic subtypes of Stargardt macular dystrophy-fundus flavimaculatus. *Arch. Ophthalmol.* **2001**, *119*, 359–369.
9. Fishman, G.A. Fundus flavimaculatus. A clinical classification. *Arch. Ophthalmol.* **1976**, *94*, 2061–2067.
10. Duncker, T.; Marsiglia, M.; Lee, W.; Zernant, J.; Tsang, S.H.; Allikmets, R.; Greenstein, V.C.; Sparrow, J.R. Correlations among near-infrared and short-wavelength autofluorescence and spectral-domain optical coherence tomography in recessive Stargardt disease. *Invest. Ophthalmol. Vis. Sci.* **2014**, *55*, 8134–8143.
11. Cideciyan, A.V.; Swider, M.; Aleman, T.S.; Sumaroka, A.; Schwartz, S.B.; Roman, M.I.; Milam, A.H.; Bennett, J.; Stone, E.M.; Jacobson, S.G. ABCA4-associated retinal degenerations spare structure and function of the human parapapillary retina. *Invest. Ophthalmol. Vis. Sci.* **2005**, *46*, 4739–4746.
12. Fujinami, K.; Sergouniotis, P.I.; Davidson, A.E.; Wright, G.; Chana, R.K.; Tsunoda, K.; Tsubota, K.; Egan, C.A.; Robson, A.G.; Moore, A.T.; et al. Clinical and molecular analysis of Stargardt disease with preserved foveal structure and function. *Am. J. Ophthalmol.* **2013**, *156*, 487-501.e1.
